# The Impact of Obesity on Cardiovascular Fitness in Young Individuals

**DOI:** 10.7759/cureus.29060

**Published:** 2022-09-11

**Authors:** Prajakta Radke, Sheela Bargal, Swati Sonawane

**Affiliations:** 1 Department of Physiology, Dr. D.Y. Patil University, School of Medicine, Navi Mumbai, IND; 2 Department of Forensic Medicine and Toxicology, Dr. D.Y. Patil University, School of Medicine, Navi Mumbai, IND

**Keywords:** harvard fitness index (hfi), cardiovascular diseases (cvd)., waist to hip ratio (whr), body mass index (bmi), obesity

## Abstract

Background

Young individuals are often at a higher risk for cardiovascular disease and obesity due to lifestyle changes like less physical activity and a sedentary lifestyle.

Objective

The aim of this study is to determine cardiovascular fitness in young individuals and to study the effects of obesity on their cardiovascular fitness.

Material and methods

In this study, 100 young individuals, out of which 50 were individuals with obesity and 50 were controls, including males and females, of the age group 18-25 years were included. Cardiovascular fitness was assessed in them using body mass index (BMI) and waist-to-hip ratio (WHR). Parameters like SBP (systolic blood pressure), DBP (diastolic blood pressure), PR (pulse rate), and HFI (Harvard fitness index) were measured.

Results

There was no difference found in the PR of the group with obesity compared to the control group (79.020/min ± 8.651 versus 79.42/min ± 6.737; p value = 0.797). However, a significant increase was observed in both SBP and DBP amongst the group with obesity compared to the control group (SBP = 122.72 mmHg ± 12.287 versus 110.92 mmHg ± 11.803; p-value < 0.001, DBP = 81.96 mmHg ± 7.913 versus 73.24 mmHg ± 11.06; p-value < 0.001). There was a significant reduction in HFI in the group with obesity than in the control group (57.44% ± 9.322 versus 80.34% ± 12.594; p-value < 0.001). When we compared males with obesity and females with obesity, we observed a non-significant difference in PR between males with obesity and females with obesity (77.12/min ± 6.02 versus 80.92/min ± 10.44; p-value = 0.122). However, we found a significant increase in SBP in males with obesity compared to females with obesity (127.76 mmHg ± 10.93 versus 117.68 mmHg ± 11.66; p-value < 0.01). A significant decrease in DBP in males with obesity (78.80 mmHg ± 7.55 versus 85.12 mmHg ± 7.07; < 0.01) than in females with obesity was also observed. Along with a non-significant increase in HFI value in males with obesity compared to females with obesity (58.96% ± 8.14 versus 55.92% ± 10.31; p-value = 0.253).

Conclusion

Results suggest that both male and female young individuals with obesity are at higher risk for developing cardiovascular comorbidities in the future. So, we need to focus on encouraging activities that promote physical fitness.

## Introduction

Obesity is a public health concern for both developed and developing nations due to the increased risk of chronic diseases such as cardiovascular diseases, stroke, diabetes, sleep apnea, osteoarthritis, and different types of cancer [1-4]. According to WHO, in 2016, 39% of adults aged 18 years and above were overweight, while 13% suffered from obesity. Globally approximately 2.8 million deaths are reported due to being overweight or obese [1]. Unfavorable cardiovascular risk is found in the young because of lifestyle changes like less physical activity, sedentary lifestyle, stress and intake of commercial food. As a result, there is low cardiovascular fitness and a high percentage of body fat in them [5]. With the advancement in technology, the prevalence of obesity has increased drastically among children and teenagers worldwide. It is an important health issue, considering that the probability of adolescents with obesity becoming adults with obesity is around 83% [6].

Obesity is a multi-factorial metabolic disease influenced by social, physiological, metabolic, molecular, and genetic factors [7]. WHO defines overweight and obesity as abnormal and excessive fat accumulation that presents a risk to health. BMI appears to be one of the indices of obesity as it approximates adiposity and fat distribution in adults [8,9]. The waist-to-hip ratio is also an important indicator of obesity [10,11]. In the present study, BMI values, decided per the guidelines issued by the Indian health ministry, and waist-to-hip ratio were used to decide obesity. Cardiovascular parameters like PR (pulse rate), BP (blood pressure), and HFI (Harvard fitness index) were also studied among the group with obesity and the control. HFI is a cardiac stress test often used for detecting and diagnosing cardiovascular disease. It is a decent way of measuring fitness and an individual’s ability to recover after strenuous exercise by checking the recovery rate [12,13].

Therefore, this research was undertaken to explore the effect of obesity on cardiovascular fitness in young individuals with and without obesity and compare cardiac fitness between males with obesity and females with obesity.

## Materials and methods

This is a cross-sectional study that was conducted on 100 young individuals - 50 with obesity and 50 as control, including both males and females aged 18-25 years from a medical college. Body mass index and waist-to-hip ratio are the two measurements we have used to determine obesity in individuals. Also, weight and height were measured, and BMI was calculated by the formula: BMI = Weight (Kg)/Height (m2) [7]. Waist circumference was measured at the level of the umbilicus, and hip circumference was measured around the widest portion of the buttocks using a measuring tape with light clothing. The waist-to-hip ratio was calculated as WHR = Waist Circumference/Hip Circumference [7,14,15]. Those individuals with BMI ≥ 25, WHR > 0.90 in males, and WHR > 0.85 in females, were considered as a group with obesity. And the individuals with BMI <23, WHR ≤ 0.90 in males, and WHR ≤ 0.85 in females, were taken as the control group. They were classified according to the cut-off values of BMI for Indians as per the guidelines issued by the Indian Health Ministry and based on WHR values as per WHO guidelines [7,14,15].

Individuals with a BMI of 23 and 24 (overweight) were excluded from the study [16]. Students with a history of diabetes, hypertension, lung disease, or taking any medications that can affect the cardiovascular and respiratory systems, alcohol intake, smoking and regularly exercising, physically handicapped, and women in the menstruating phase were also excluded from this study. After the selection of the groups with obesity and the control group, the study participants refrained from any energetic physical activity for 2 to 3 hours before the test. Moreover, the following parameters were studied amongst the study participants at room temperature between 1 pm and 4 pm a) Resting pulse rate was calculated by counting radial pulse for 1 minute; b) Blood pressure was measured by the auscultatory method using a Mercury Sphygmomanometer in a sitting position after 10 min; c) Harvard fitness index (%): Calculated using the Harvard step test, metronome, and stopwatch. The person steps up and down on Harvard Step, 20 inches high, at the rate of 30 steps per minute for 5 minutes or until exhaustion. The person immediately sits down on completion of the test, and the total number of pulse beats was counted and recorded between 1 and 1.5 minutes (PR1), between 2 and 2 .5 minutes (PR2), and between 3 and 3.5 minutes (PR3). The Harvard fitness index is calculated using the following formula HFI = Duration exercise (in sec.) X 100/ 2 (PR1 + PR2 + PR3) [12].

Cardiovascular fitness is classified according to the Harvard fitness index as follows: HFI below 55 is considered as poor fitness, 55-64 as low average fitness, 65-79 as high average fitness, 80-89 as a good fitness index, and 90 and above as excellent fitness [12].

Data analysis was done using SPSS version 17.0 software (SPSS Inc., Chicago, USA), and further analysis was performed using an unpaired t-test.

Permission to conduct the study was taken from the Institutional Ethics Review Committee, Mahatma Gandhi Mission’s (MGM) Institute of Health Sciences, (Deemed University u/s 3 of the UGC Act, 1956) Kamothe, Navi Mumbai, India - Institutional Ethics Review Committee (IERC) approval number: MGM/HIS/RS/2011/26.

## Results

It was observed that the mean value of pulse rate of the group with obesity (79.020/min ± 8.651) was lower than the control group (79.420/min ± 6.737), which was statistically not significant. The results indicated significantly higher mean values of SBP and DBP in the group with obesity (122.720 mmHg ± 12.287 and 81.960 mmHg ± 7.913) than in the control group (110.920 mmHg ± 11.803 and 73.240 mmHg ± 11.065). For HFI, significantly lower values were observed in the group with obesity (57.440 % ± 9.322) compared to the control group (80.340% ± 12.594) (Table 1).

**Table 1:** Cardiovascular parameters in the obese group and the control group.

Parameters	Group	Mean± SD	p-value
Pulse rate/minute	Obese	79.020 ± 8.651	0.797
	Control	79.420 ± 6.737	
Systolic blood pressure in mm/Hg	Obese	122.720 ± 12.287	< 0.001
	Control	110.920 ± 11.803	
Diastolic blood pressure in mm/Hg	Obese	81.960 ± 7.913	< 0.001
	Control	73.240 ± 11.065	
Harvard fitness index	Obese	57.440 ± 9.322	< 0.001
	Control	80.340 ± 12.594	

When we compared males with obesity and females with obesity, PR was slightly higher in females with obesity (80.92/min ± 10.44) than in males with obesity (77.12/min ± 6.02) which was statistically not significant. The mean value of SBP (systolic blood pressure) was significantly higher in males with obesity (127.76 mmHg ± 10.93) than in females with obesity (117.68 mmHg ± 11.66), and the mean value of DBP (diastolic blood pressure) was significantly higher in females with obesity (85.120 mmHg ± 7.07) than in males with obesity (78.80 mmHg ± 7.55). In females with obesity, the HFI values (55.92% ± 10.31) were slightly lower than in males with obesity (58.96% ± 8.14) which were statistically not significant (Table 2).

**Table 2:** Cardiovascular parameters in obese male and obese females

Parameters	Obese Male	Obese Female	
	Mean ± SD	Mean ± SD	p-value
Pulse rate/minute	77.12 ± 6.02	80.92 ± 10.44	0.122
Systolic blood pressure in mm/Hg	127.76 ± 10.93	117.68 ± 11.66	<0.01
Diastolic blood pressure in mm/Hg	78.80 ± 7.55	85.120 ± 7.07	<0.01
Harvard fitness index	58.96 ± 8.14	55.92 ± 10.31	0.253

## Discussion

This study was designed to evaluate cardiovascular fitness in young individuals with obesity. We found a significant increase in SBP, DBP, and a significant reduction in HFI in individuals with obesity but no difference was found in PR in young individuals with obesity compared to those without obesity. It implies that young individuals with obesity are at higher risk for cardiovascular disease in the future. When we compared males with obesity with females with obesity, we observed a non-significant difference in PR and HFI values. In contrast, a significant increase in SBP in males with obesity and a significant increase in DBP in females with obesity was observed. So, male and female young individuals are at the same risk where obesity is concerned, as SBP was higher in males, but DBP was higher in females. Various similar studies showed a non-significant reduction in PR in individuals with obesity [17,18]. A study by Ofuya et al. showed a significant increase in PR in individuals with obesity [19]. A similar significant increase in BP among individuals with obesity was observed in various studies [20,21]. Additionally, a similar significant decrease in HFI in individuals with obesity was observed in studies conducted by Chen et al. and Dasa et al. [13,22].

Our study supports that young individuals with obesity are at higher risk for cardiovascular disease. This is due to their lifestyle changes like less physical activity and more sedentary lifestyle. [1,4]. The American Heart Association has cited obesity as a major modifiable risk factor for coronary heart disease. People with obesity are likely to have hypertriglyceridemia and low HDL (high-density lipoprotein) cholesterol values, which increases the risk of cardiovascular disease. Also, it was found that obesity is associated with insulin resistance and hyperinsulinemia. Excess insulin causes sodium retention, blood volume expansion, and the production of norepinephrine in the body, resulting in hypertension in individuals with obesity [23]. Being overweight and having hypertension ultimately lead to the development of cardiovascular diseases in individuals with obesity. Cardiac weight increases with increasing body weight, suggesting increased cardiac work, which results in cardiomyopathy and heart failure [24]. This indicates that obesity affects cardiovascular function to a greater extent. The present study supports this, indicating a significant decrease in cardiovascular function in young individuals with obesity than in those without obesity. So, we need to pay more attention to the health of young individuals and need to improve their physical fitness.

The strength of our study is that we have included two criteria to assess obesity: 1) BMI and 2) WHR. young individuals who fulfil both criteria were included in this study. As BMI is not gender-specific and independent of age and regional distribution of fat, excess weight may come from muscles, bone, fat, or body water. BMI overestimates the body fat in a person who is very muscular or has fluid retention and underestimates the body fat in a person who has lost muscle mass, as in an elderly person. BMI is a poor measure of abdominal obesity, which is an independent risk factor for cardiovascular disease [25]. The waist-to-hip ratio is commonly used to judge abdominal obesity and it is more gender-specific and population-specific, so we have included it in our study to assess obesity along with BMI [26]. 

The limitations of our study are in terms of the number and nature of participants. In future studies, we can increase the sample size and study cardiovascular fitness in children with obesity. In future studies, we can also use VO2 max as an indicator of cardiovascular fitness. We can also evaluate respiratory parameters to study respiratory fitness in young individuals with obesity.

## Conclusions

Low cardiovascular fitness in young adults with increased body fat could be a factor in developing cardiovascular comorbidities like coronary artery disease and stroke later in life. Results suggest that young individuals with obesity are at a higher risk for developing cardiovascular comorbidities later in life than young individuals without obesity. Males with obesity and females with obesity are at the same risk as far as obesity is concerned. Exercise training and healthy eating habits are preventive measures for cardiovascular diseases. Our study suggests that young individuals need regular physical exercise to prevent obesity and related cardiovascular risk in the future.

## References

[1] Sonawane S, Kadam A, Sharma H, Tetarbe T (2019). To study the prevalence of obesity among medical students in relation with dietary habits. Ind J Forens Communit Med.

[2] Islam M, Haque M (2020). Measuring the association of overweight and obesity with human disease and other factors in Bangladesh. Open J Stat.

[3] El-Sherbiny NA, Mashahit MA, Sheir RE (2014). Assessment of public awareness about body measurements among Fayoum population. Health.

[4] Tejada T, Pastor D, Gaytan H, Norma A, Ortiz Ortiz (2015). Prevalence and factors associated with overweight and obesity among university students of the health field in San Luis Potosí México. Health.

[5] Snel GJ, van den Boomen M, Hurtado-Ortiz K (2022). Cardiac alterations on 3T MRI in young adults with sedentary lifestyle-related risk factors. Front Cardiovasc Med.

[6] Saavedra JM (2014). Obesity - a risk factor or a disease: What can exercise do for obese children?. Indian J Med Res.

[7] Dr A. Rastogi (2022). Obesity | National Health Portal of India. nhp.gov.in.

[8] Chaudhary S, Kang MK, Sandhu JS (2010). The effects of aerobic versus resistance training on cardiovascular fitness in obese sedentary females. Asian J Sports Med.

[9] Lim KG (2016). A review of adult obesity research in Malaysia. Med J Malaysia.

[10] Badaruddoza Badaruddoza, Kaur N. Basanti B (2021). Research on Inter-relationship of waist-to-hip ratio, body mass index and subcutaneous fat with blood pressure among university-going Punjabi Sikh and Hindu females. Afr J Nurs and Midwife.

[11] Ahmad N, Adam SI, Nawi AM, Hassan MR, Ghazi HF (2016). Abdominal Obesity Indicators: Waist Circumference or Waist-to-hip Ratio in Malaysian Adults Population. Int J Prev Med.

[12] Bora R, Dutta U (2020). To study the effect of exercise on physical fitness of healthy medical students and trained athletes using step test. Indian journal of applied research.

[13] Dasa B, Ghosh T, Gangopadhyay S (2010). A Comparative study of physical fitness index (PFI) and predicted maximum aerobic capacity (VO2max) among the different groups of female students in West Bengal, India. Int J App Sport Sci.

[14] Baisakhiya S, Singh S, Manjhi P (2016). Correlation between age, gender, waist-hip ratio and intra ocular pressure in Adult North Indian population. J Clin Diagn Res.

[15] Singh N, Hooja H, Yadav A, Mital P, Jaiswal A, Bairwa P (2022). Waist circumference and waist-to-hip ratio as indicators of abdominal obesity. Int J Life Sci Res Arch.

[16] Pawalia A, Yadav V, Kulandaivelan S (2015). Effect of obesity on pregnancy outcomes - Indian perspective: a review. Int J Sci Res.

[17] Ozcelik O, Aslan M, Ayar A, Kelestimur H (2004). Effects of body mass index on maximal work production capacity and aerobic fitness during incremental exercise. Physiol Res.

[18] So WY, Choi DH (2010). Differences in physical fitness and cardiovascular function depend on BMI in Korean Men. J Sports Sci Med.

[19] Ofuya ZM, Georgewill AA, Agu GO (2005). A Study of cardiovascular and respiratory parameters in obese and non-obese subjects’ resident in Port Harcourt. African Journal of Applied Zoology & Environmental Biology.

[20] Chorin E, Hassidim A, Hartal M, Havakuk O, Flint N, Ziv-Baran T, Arbel Y (2015). Trends in adolescents obesity and the association between BMI and blood pressure: a cross-sectional study in 714,922 healthy teenagers. Am J Hypertens.

[21] Thompson P, Logan I, Tomson C, Sheerin N, Ellam T (2020). Obesity, sex, race, and early onset hypertension: implications for a refined investigation strategy. Hypertension.

[22] Chen LJ, Fox KR, Haase A, Wang JM (2006). Obesity, fitness and health in Taiwanese children and adolescents. Eur J Clin Nutr.

[23] Zanovec M, Lakkakula A, Johnson L, Turni G (2009). Physical activity is associated with percent body fat and body composition and body mass index in white and black college students. Int J Exerc Sci.

[24] Bray GA (2004). Medical consequences of obesity. J Clin Endocrinol Metabol.

[25] Zhang C, Rexrode KM, van Dam RM, Li TY, Hu FB (2008). Abdominal obesity and the risk of all-cause, cardiovascular, and cancer mortality: sixteen years of follow-up in US women. Circulation.

[26] Karen O (2022). Is BMI the Best Measure for Obesity | Cancer Research Catalyst | American Association for Cancer Research. Cancer Research Catalyst.

